# Data mining of plasma peptide chromatograms for biomarkers of air contaminant exposures

**DOI:** 10.1186/1477-5956-6-6

**Published:** 2008-01-30

**Authors:** Subramanian Karthikeyan, Premkumari Kumarathasan, Renaud Vincent

**Affiliations:** 1 Safe Environments Program, Healthy Environments and Consumer Safety Branch, Health Canada, 0803C Tunney's Pasture, Ottawa, Ontario, K1A 0K9, Canada

## Abstract

**Background:**

Interrogation of chromatographic data for biomarker discovery becomes a tedious task due to stochastic variability in retention times arising from solvent and column performance. The difficulty is further compounded when the effects of exposure (e.g. to environmental contaminants) and biological variability result in varying numbers and intensities of peaks among chromatograms.

**Results:**

We developed a software tool to correct the stochastic time shifts in chromatographic data through iterative selection of landmark peaks and isometric interpolation to improve alignment of all chromatographic peaks. To illustrate application of the tool, plasma peptides from Fischer rats exposed for 4 h to clean air or Ottawa urban particles (EHC-93) were separated by HPLC with autofluorescence detection, and the retention time shifts between chromatograms were corrected (dewarped). Both dewarped and non-dewarped datasets were then mined for models containing peptide peaks that best discriminate among the treatment groups using ClinproTools™. In general, models generated by dewarped datasets were able to better classify test sample chromatograms into either clean air or EHC-93 exposure groups, and 0 or 24 h post-recovery time groups. Peak areas of peptides in a model that produced the best discrimination of treatment groups were analyzed by two-way ANOVA with exposure (clean air, EHC-93) and recovery time (0 h, 24 h) as factors. Statistically significant (p < 0.05) time-dependent and exposure-dependent increases and decreases were noted establishing these as biomarker candidates for further validation.

**Conclusion:**

Our software tool provides a simple and portable approach for alignment of chromatograms with complex, bi-directional retention time shifts prior to data mining. Reliable biomarker discovery can be achieved through chromatographic dewarping using our software followed by pattern recognition by commercial data mining applications.

## Background

Biomarkers are of interest and value in many experimental population studies and in clinical assessments as biological indicators of environmental exposures or disease status [1-4]. Detection of selective and sensitive markers, however, requires appropriate choice and application of experimental and data analysis approaches. While modern analytical tools allow simultaneous measurements of many genes, proteins or metabolites, mining of these enormous and often complex datasets for sensitive, specific and biologically relevant changes requires approaches that not only handle data complexity, in order to derive meaningful inferences, but also permit correcting for the underlying distortions or discrepancies in the datasets, arising from the nature of the analyses or analytical platform [5-8]. For example, in the case of liquid chromatography, an integral component of several proteomics platforms, retention time shifts across a series of chromatographic runs can arise due to changes in quality of mobile phases or column performance. This makes the data less amenable for direct differential comparisons of treatment responses or pattern recognition for sample source identification or exposure determination [9].

Various approaches of peak alignment such as local shifting and peak matching [10], target peak alignment [9,11], rank alignment [12], correlation optimized warping [9,13-15], dynamic time dewarping [16], parametric time warping [15,17], semi-parametric time warping [9,15] and fuzzy warping [18] have been applied to dewarp liquid or gas chromatograms with varying degrees of efficiency. A comparison of correlation optimized warping, target peak alignment and semi-parametric time warping for alignment of chromatograms concluded that alignment based on semi-parametric time warping differed from that of the other two approaches [9]. However, the aligned datasets from all three methods resulted in similar score plots during post-processing of data by principal component analysis (PCA) and therefore led to an equivalent discrimination of the samples. Another study [15] comparing correlation optimized warping, parametric time warping and semi-parametric time warping concluded that parametric time warping supported alignment of only non-complex peak shifts that occur in both directions, whereas correlation optimized warping and semi-parametric time warping were able to correct complex bi-directional peak shifts, however, at the expense of a need to optimize input parameters (e.g. the warping function and a penalty parameter for semi-parametric time warping, and the section length and slack for correlation optimized warping). Tomasi et al. [19] compared correlation optimized warping and dynamic time warping as pre-processing methods for chromatographic datasets prior to analysis by PCA and concluded that dynamic time warping with rigid slope constraints and correlation optimized warping were superior to unconstrained dynamic time warping. These results suggest that in addition to the demonstrated differences in the efficiency and applicability of these approaches to specific chromatographic datasets, there is also a requirement for the definition and experimentation with a number of dewarping parameters to obtain good alignments. All of the above algorithms are routinely implemented in a mathematical computing environment (e.g. subroutines on MatLab).

The objective of this work was to develop and validate a dewarping approach that is implemented in a simple and portable software tool, and that is not constrained by a need to specify or optimize dewarping parameters, to correct the stochastic variability in chromatographic data manifested as large complex bi-directional retention time shifts and to generate datasets better amenable to downstream data mining for biomarkers.

## Results

### Implementation and validation of dewarping approach

**Figure 1:**
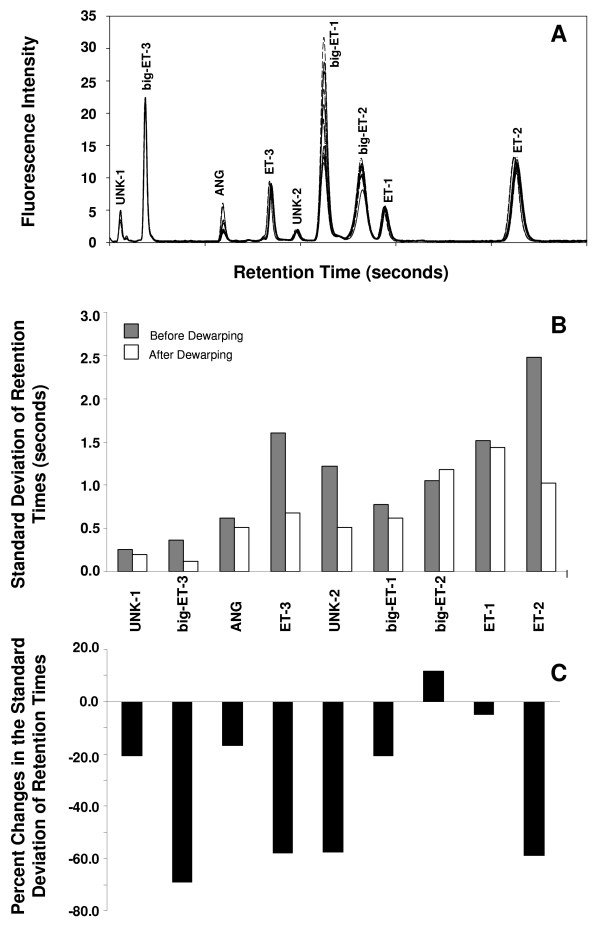
**Validation of dewarping using our approach**. An overlay of chromatograms from 12 consecutive runs of a standard analyte mixture shows the small retention time shifts that were observed between runs (A). Reduction of variation in the retention times of each peak when the remaining peaks were aligned by dewarping, expressed as standard deviation in seconds (B), and as % changes in standard deviation (C), is also shown. Analytes that are labeled as UNK-1 and UNK-2 are unknown degradation products of the standard analytes.

Our dewarping approach consisted of a software assisted iterative selection and alignment of landmark peaks by interpolation or removal of data points between peaks for dewarping of all peaks across chromatograms. This was implemented as a Windows based software tool ("DewarpTool"). Dewarping using this approach was validated by the application of the tool to dewarp chromatograms from 12 successive runs of a mixture of standard peptides. An overlay of standard chromatograms (Figure 1A) illustrates the minor shifts in retention times that existed before dewarping. Dewarping of the chromatograms reduced retention time variations, expressed as standard deviation in seconds, for all analytes except for big ET-2 (Figure 1B). For big ET-3, ET-3, UNK-2 and ET-2, the variations were reduced by >50% with the largest reduction (69%) noted for big ET-3 (Figure 1C).

**Figure 2:**
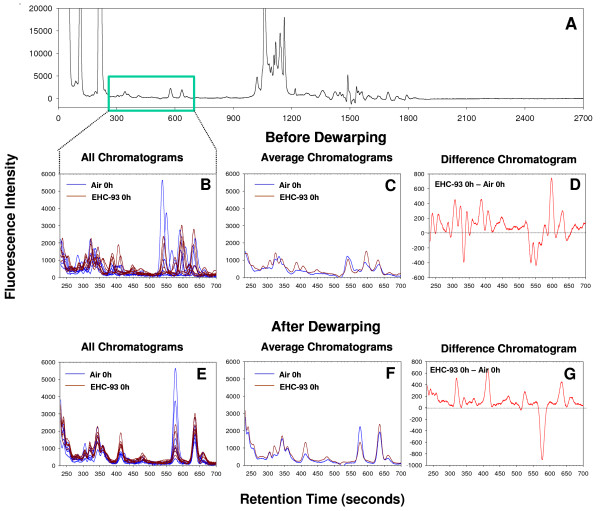
**Panel showing that peak alignment by dewarping facilitates investigation of differential responses**. A typical plasma chromatogram is shown (A). Sections (250–700 seconds) of plasma peptide chromatograms from rats exposed to clean air and EHC-93 illustrate peak shifts that occurred before dewarping (B) and improved peak alignment by dewarping (E). Better alignment of peaks in dewarped chromatograms resulted in more reliable average chromatograms (F) than those obtained with non-dewarped chromatograms (C), for differential comparisons. The difference chromatograms further facilitate visualization of treatment effects (D and G).

Retention time variations were also seen in complex plasma peptide chromatograms from animals exposed to clean air and to Ottawa urban particles (EHC-93) by inhalation. A representative chromatogram of the plasma peptide profile recovered for rat plasma across the entire run (2700 seconds or 45 min) is shown in Figure 2A. The retention time variability in rat plasma chromatograms (clean air, n = 6; EHC-93, n = 6), as seen in a small section of the chromatograms corresponding to 240 seconds to 700 seconds of run after the start (Figure 2B), was relatively larger compared to that of the standard analyte chromatograms (Figure 1A). For some of these peptide peaks, the retention times varied as much as 46 seconds (data not shown). Iterative dewarping improved alignment of plasma peptide peaks resulting in chromatograms in which the treatment effects on individual peptides were easily discernible (Figure 2E). Averages of non-dewarped chromatograms showed dubious and poorly aligned peaks, and were difficult to compare (Figure 2C), and some of the treatment responses shown by the difference trace between non-dewarped average chromatograms (Figure 2D) were likely due to peak misalignments. In contrast, dewarped spectra resulted in well-aligned average chromatograms (Figure 2F) amenable for comparisons by differential subtraction with the differential responses (Figure 2G) being consistent with the changes noted in the average chromatograms.

### Data mining of dewarped and non-dewarped data

**Figure 3:**
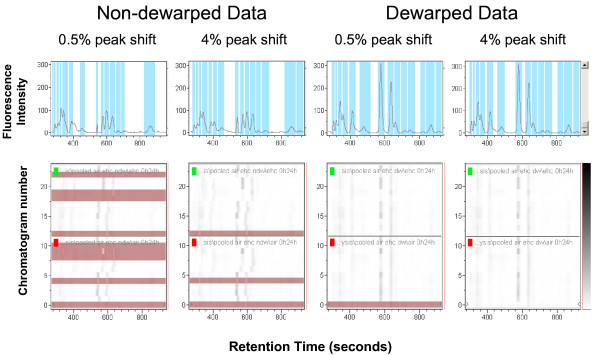
**Effects of dewarping on recalibration by ClinproTools**. Both dewarped and non-dewarped plasma peptide chromatograms were mined by ClinproTools. Shown is a comparison of the two exposure groups (air exposure with 0 or 24 h recovery post-exposure, n = 12; EHC-93 exposure with 0 or 24 h recovery post-exposure, n = 12). Poor alignment of peaks in the non-dewarped datasets rendered many chromatograms non-recalibratable (highlighted in the gel-view), and therefore less reliable for use in biomarker analysis. In addition, the peptide profiles of the total average chromatograms were less impacted by the magnitude of peak shift window when using dewarped datasets (spectral view).

In order to examine if our dewarping approach generated chromatograms that are better amenable to data mining for generation of discriminatory models, both dewarped and non-dewarped chromatograms were mined using ClinproTools. While a number of non-dewarped chromatograms were not calibratable and therefore unusable for discriminatory model generation even with a large maximal peak shift parameter to accommodate large peak shifts (Figure 3), the dewarped datasets were generally more amenable for data mining using the multivariate approaches, the genetic algorithm (GA) and support vector machine (SVM) followed by K-nearest neighbors classification (KNN) for evaluation of discriminatory capacity. The total average chromatograms generated using non-dewarped datasets were also impacted by the changes to the peak shift parameter (0.5% or 4.0%) made in order to maximize the number of recalibratable and, therefore, useable chromatograms. In contrast, total average chromatograms of dewarped datasets were not impacted by the magnitude of the peak shift parameter.

**Figure 4:**
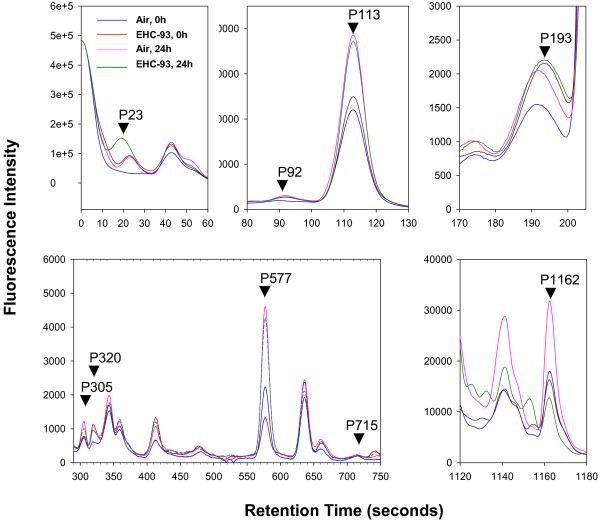
**Candidate markers in the best-discriminatory model generated by ClinproTools**. Markers in a model that best discriminated between the treatment groups (air and EHC-93 exposures with 0 h or 24 h recovery). Chromatographic regions containing these peaks are shown to illustrate the treatment effects.

**Figure 5:**
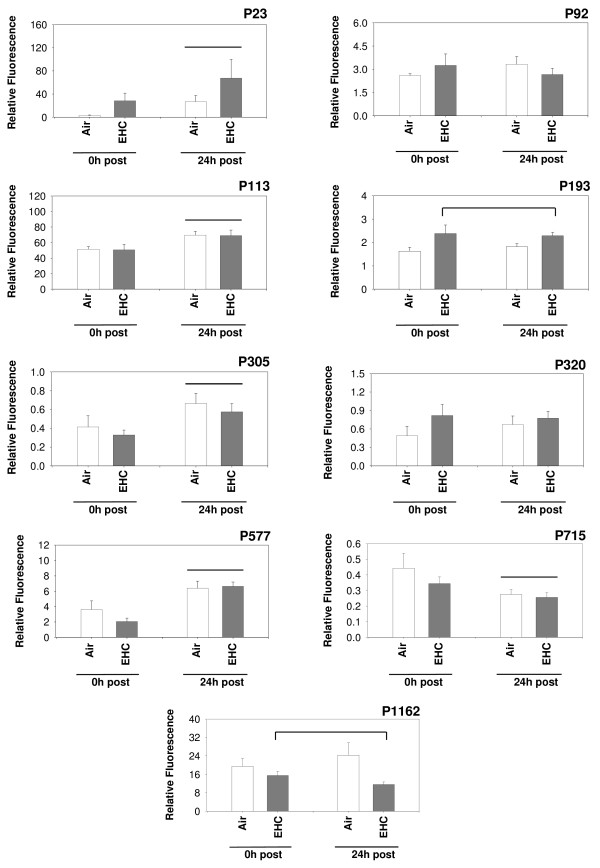
**Treatment effects on candidate markers**. Two-way ANOVA of peak areas of the candidate markers, with time (0 h, 24 h) and exposure (clean air, EHC-93) as factors, showed significant time-dependent increases (0 h<24 h, p < 0.05) in P23, P113, P305 and P577 as well as a time-dependent decrease (0 h>24 h, p < 0.05) in P 715. Significant (p < 0.05) particle exposure effects were noted for P193 (air<EHC-93) and P1162 (air>EHC-93).

Discriminatory models generated using both dewarped and non-dewarped datasets (after excluding the non-recalibratable spectra) were validated by their ability to classify experimental samples into air or EHC-93 exposure groups (irrespective of duration of recovery; exposure effects) and into 0 and 24 h post-recovery time groups (irrespective of exposure; time effects). In general, models generated using dewarped datasets had a better discriminatory capacity when compared to the models generated by using non-dewarped datasets (Table 1). Among these, the GA-generated model provided the best discrimination between the treatment groups, resulting in a classification efficiency 100% for air exposures, 100% for EHC exposures, 91.7% for 0 h post-exposure recovery, and 91.7% for 24 h post-exposure recovery. This model consisted of 9 marker peaks: P23, P92, P113, P193, P305, P320, P577, P715 and P1163 (Figure 4). A separate two-way ANOVA of the areas of these marker peaks, with time (0 h, 24 h) and exposure (clean air, EHC-93) as factors, showed significant (p < 0.05) time of recovery effects: 0 h < 24 h in P23, P113, P305 and P577, and 0 h > 24 h in P715 (Figure 5). Significant (p < 0.05) EHC-93 exposure-dependent changes included an increase in the levels of P193 (air<EHC-93) and a decrease in levels of P1162. A non-significant EHC-93 dependent increase was noted for P320.

**Table 1:** Percent true classification of chromatographic data by discriminatory models

Treatment Groups	Expected Classification	Dataset	Percent True Classification by Models	
			GA	SVM

Clean air, 0 h and 24 h recovery post-exposure	Clean air	dewarped	100.00	100.00
		non-dewarped	91.67	83.33
EHC-93, 0 h and 24 h recovery post-exposure	EHC-93	dewarped	100.00	83.33
		non-dewarped	66.67	83.33
0 h recovery post- exposure (Clean air and EHC-93)	0 h	dewarped	91.67	100.00
		non-dewarped	75.00	91.67
24 h recovery post- exposure (Clean air and EHC-93)	24 h	dewarped	91.67	91.67
		non-dewarped	75.00	91.67

Percent true classification of all chromatographic data (dewarped or non-dewarped) into exposure (clean air and EHC-93) and post-exposure recovery time (0 h, 24 h) groups by discriminatory models based on the genetic algorithm (GA) and the support vector machine algorithm (SVM).

The results of cross-validation of models using the approaches the One-Out, Random and K-Folds, wherein one or more of the chromatograms were randomly or sequentially excluded from model generation and were classified against models generated using the remaining data as the training data, are shown in Table 2. The values shown are averages of a number of iterations of testing, wherein the excluded chromatograms served as test data. Cross validation results also showed that dewarped datasets generated models that were better able to discriminate between exposures (EHC-93 and clean air) or post-exposure times of recovery (0 h and 24 h). Non-dewarped data with 0.5% peak shift window did not allow cross validation, as a number of chromatograms using this window were not recalibratable.

**Table 2:** Predictive capacity of models as determined by cross-validation

**Classification**	**Dataset**	**Validation method**	**0.50% peak shift window**		**4.0% peak shift window**	
			
			**GA**	**SVM**	**GA**	**SVM**
Air vs. EHC-93	dewarped	One out	78.41	70.08	79.17	66.67
		Random	65.83	60.83	71.51	73.33
		K-folds	73.86	61.36	70.83	70.83
	non-dewarped	One out	*a*	*a*	57.73	52.23
		Random	*a*	*a*	61.46	55.21
		K-folds	*a*	*a*	47.73	48.64
0 h vs. 24 h	dewarped	One out	72.83	81.82	66.67	87.50
		Random	72.92	76.04	58.33	87.50
		K-folds	72.73	72.73	81.44	83.58
	non-dewarped	One out	*a*	*a*	54.55	72.73
		Random	*a*	*a*	68.75	66.67
		K-folds	*a*	*a*	63.64	72.73

Reliability (future predictive capacity) of discriminatory models generated using dewarped and non-dewarped datasets as determined by cross validation using the One-out, the Random or the K-folds methods. Values provided are the percent averages of the predictive capacities of a number of models generated by different combinations of chromatograms as training (model generation) and test (model validation) data. GA and SVM were used in model generation. Cross validation was not possible when there was an insufficient number of recalibratable chromatograms within an exposure or time of recovery group, and is indicated by a '*a*'.

## Discussion

The need to have well aligned chromatographic fingerprints for data mining and visualization approaches such as PCA has been underlined in many previous studies. Examples include: use of correlation optimized warping of GC-MS data followed by PCA for fingerprinting of petroleum biomarkers [20], correlation optimized warping of fast HPLC chromatograms followed by partial least squares (PLS) and uninformative variable elimination partial least squares (UVE-PLS) to construct multivariate regression models to predict total antioxidant capacity of tea extracts [14], and fuzzy warping of HPLC peptide profiles followed by PCA, multi-dimensional scaling (MDS) and cluster analysis for chemometric analysis of cheese extracts [21]. Our work describes a new alignment approach, successfully validated, implemented and used to generate liquid chromatographic datasets amenable for data mining of peptide profiles for biomarkers of pollutant exposure and effects.

Our alignment approach consists of software-aided, visual matching of landmark peaks across chromatograms, followed by programmatic, isometric interpolation or removal of data points, to stretch or shrink regions between landmark peaks to achieve their alignment. Johnson et al [10] employed an automated peak matching algorithm also based on local shifting and peak matching by interpolation of data points for alignment of gas chromatograms, but the alignment process required that corresponding peaks in the target and sample chromatograms were not offset in retention time by more than the typical distance between adjacent peaks. The larger magnitude of shifts (up to 46 seconds) compared to inter-peak distances (as small as 20 seconds) in our liquid chromatograms were clearly not amenable to such an approach. Target peak alignment proposed by Xu et al [11] also involves stretching and shrinking of chromatographic regions to achieve peak matching, however, it requires a window-target-test factor analysis to define matching peaks. Our visual peak matching and programmatic dewarping approach provides simplicity and greater reliability for correction of large, complex retention time shifts in chromatograms with unequal number of peaks, and large sample-to-sample differences in peak areas, resulting either from biological variability or from treatment effects.

The correction of retention time shifts in our plasma peptide chromatograms resulted in datasets amenable for averaging and differential comparisons using the differential analysis component of DewarpTool. Although this allowed a visual verification of chromatogram alignment and a qualitative assessment of treatment-dependent responses that may warrant detailed quantitative analysis, given the potential subjectivity of a visual peak selection approach and the availability of commercial data mining tools, we tested whether our dewarping approach can generate datasets that are more amenable than the non-dewarped chromatographic data for an objective analysis of biomarkers using ClinproTools.

In order that our chromatographic data are treated appropriately within ClinproTools, a tool intended for mass spectral data mining, data preparation and peak selection parameters were appropriately adjusted for accurate definition of chromatographic peaks. The non-dewarped datasets were not as amenable to data mining as the dewarped datasets, as a larger number of non-dewarped chromatograms were not recalibratable and hence unusable for data mining even after using a large peak matching window to accommodate large retention time shifts. In addition, the choice of the peak shift parameter (0.5% or 4.0%) impacted the average peak profile generated by non-dewarped datasets, but not that of dewarped datasets, suggesting the possibility of ambiguous peak alignments by ClinproTools when using non-dewarped datasets. If this is true, it can be expected that the mining of dewarped data would generate better discriminatory models than those generated using non-dewarped datasets. This is supported by our analysis using the genetic and support vector machine algorithms, the two multivariate model-building methods provided by ClinproTools, and by examining the ability of the generated models to classify experimental chromatograms into appropriate exposure (clean air, EHC-93) and post-exposure recovery time (0, 24 h) groups. Models generated by dewarped datasets had, in general, better discriminatory capacity than those generated by non-dewarped datasets. In addition, non-dewarped datasets resulted in insufficient number of recalibratable and hence useable chromatograms for cross validation.

In short, our dewarping approach implemented in DewarpTool allowed generation of chromatographic datasets that were more suited for data mining resulting in models with better discriminatory capacity for classification of treatment groups. The peaks contained in the best discriminatory model showed both significant particle and time dependent responses and non-significant pollutant-time interactions, pointing to their potential involvement in pathways relating to air pollution related biological changes. These peptides may provide interesting marker candidates and mechanistic insights into adverse health impacts of low ambient levels of air contaminants, upon their identification. Generation of models comprising of candidate peptides showing significant treatment effects resulting in greater reliability for discrimination of treatment effects validated the models and reaffirmed the utility of our dewarping approach in generating chromatographic datasets for biomarker mining.

## Conclusion

Reduction of retention time variations of peptide peaks in rat plasma chromatograms by using DewarpTool resulted in datasets that were better amenable for data-mining and that, after mining, resulted in models of peptide peaks showing high discriminatory capacity between treatment groups (types of air pollutant exposures and times of recovery after exposures), and therefore of potential value as biomarkers after mechanistic validation. Overall, our approach involving dewarping by DewarpTool followed by mining for discriminatory biomarker patterns provides a simple, novel, portable and useful approach for the mining of liquid chromatographic data that may be characterized by large, complex peak shifts. DewarpTool installation files (Windows operating system) are provided as supplementary material to this communication [Additional files 1, 2, 3].

## Methods

### Chemical standards

The following HPLC-grade standards were used: angiotensin II acetate (human), endothelin-1 (human, porcine), endothelin-2 (human), enodothelin-3 (human, rat) and big-endothelin-2 (human) (Sigma Aldrich Canada Ltd., Oakville, ON, Canada); big-endothelin-1 (human) (Bachem Bioscience Inc., King of Prussia, PA, USA); big-endothelin-3 (rat) (Peptides International Inc., Louisville, KY, USA).

### Animals

Pathogen-free Fischer-344 male rats (180–250 g) obtained from Charles River (St. Constant, Québec, Canada) were housed in individual plexiglass cages on wood-chip bedding under HEPA-filtered air and held in a 12 h dark/light cycle. Food (laboratory rodent diet 5001; Purina Mills, LLC., St.Louis, MS, USA) and water were provided *ad libitum*. All experimental protocols were reviewed and approved by the Animal Care Committee of Health Canada. Animals were exposed for 4 hours to clean air or 50 mg/m^3 ^Ottawa urban particles (EHC-93) in nose-only exposure chambers as described previously [22]. Plasma samples were collected immediately after exposure and after 24 h recovery in clean air. Animals were anaesthetized by administration of sodium pentobarbital (60 mg/kg, ip) and blood was collected from the abdominal aorta into vacutainer tubes containing the sodium salt of ethylene diamine tetra acetic acid (EDTA) at 10 mg/ml and phenyl methyl sulfonyl fluoride (PMSF) at 1.7 mg/ml to prevent postmortem peptide changes. Plasma was separated by centrifugation (1000 g for 10 min), aliquoted, and frozen at -80°C.

### HPLC analysis

HPLC analysis of plasma peptides was conducted as described by Kumarathasan et al. [23]. Briefly, the proteins were precipitated in acid-acetone and subjected to molecular weight cut-off (30 kDa) filtration to retrieve peptides which were reconstituted in Dulbecco's phosphate-buffered saline (PBS) for HPLC separation. Stock solutions of endothelin-1, -2, and -3, and big-endothelia -1, -2 and -3 standards (0.1 mg/ml) and angiotensin II acetate (0.2 mg/ml) were prepared in PBS and stored at -20°C until use. The standard analyte mixture was prepared by mixing the stock solutions of endothelin-1, endothelin-2, endothelin-3, big endothelin-1, big endothelin-2, big endothelin-3, and angiotensin II acetate at a ratio of 4:1:3:1:4:4:3 to a total volume of 75 μl per run. HPLC of the standard analyte mixture and of rat plasma peptides was conducted in a HPLC unit consisting of a Gilson solvent delivery system (Mandel Scientific, Guelph, Ontario), a Gilson autosampler (model 231 XL; Middleton WI), a Supelcosil LC-318 reverse-phase column (25 cm length, 4.6 mm ID, 5 μm particle size, 300 Å pore dimension; Supelco, Oakville Ontario), and a Shimadzu fluorescence detector (model RF 551; Shimadzu Scientific Instruments, Columbia, MD, USA). Autofluorescence detection of peptides was performed with excitation and emission wavelengths set at 280 nm and 340 nm respectively. Chromatographic data files were exported from Unipoint System Software v.3.3 (Mandel Scientific Company Ltd., Tonawanda, New York, USA), the HPLC system operation and data management software, as tab-delimited text (.txt) files for further analyses.

### Chromatographic dewarping

**Figure 6:**
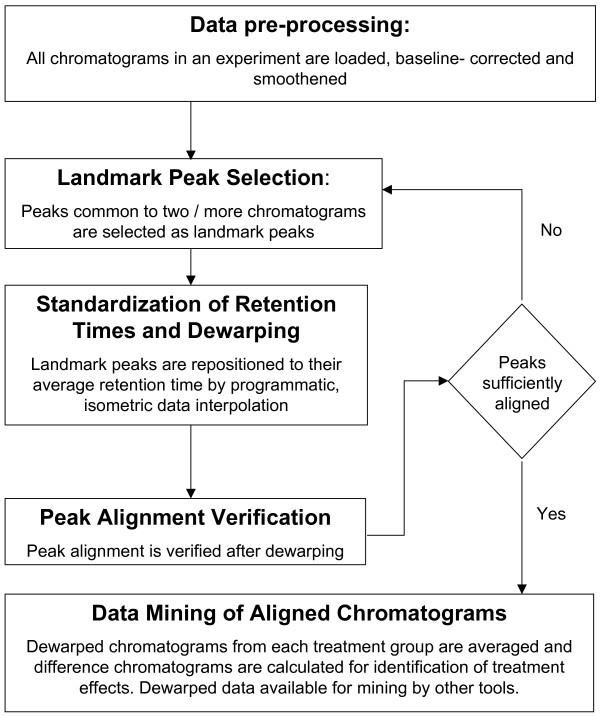
**The iterative computational approach employed in our chromatographic dewarping**. Each iteration of dewarping improves peak alignment and facilitates selection of new landmark peaks for further refinement of peak alignment. Differential comparison of average chromatograms allows a qualitative analysis of treatment responses.

We developed a dewarping software (DewarpTool), using Visual Basic (version 6.0, Professional edition, Microsoft Inc. Redmond, WA), and deployed the software in a Windows XP environment. The analysis workflow consists of baseline correction, data smoothening, landmark peak selection, peak alignment, average chromatogram calculation and differential visualization (Figure 6). Baseline correction involves calculation of the average of a defined number of fluorescence intensity data points in a chromatogram and subtracting this value from data points across the entire chromatogram. Data smoothening involves replacement of each fluorescence data point with a 3-point running average. After baseline correction and smoothening, the time axes across chromatograms are truncated to start at the chromatogram front and to contain the same number of points (e.g. 2700 seconds or 45 minutes duration in our standard and plasma chromatograms).

**Figure 7:**
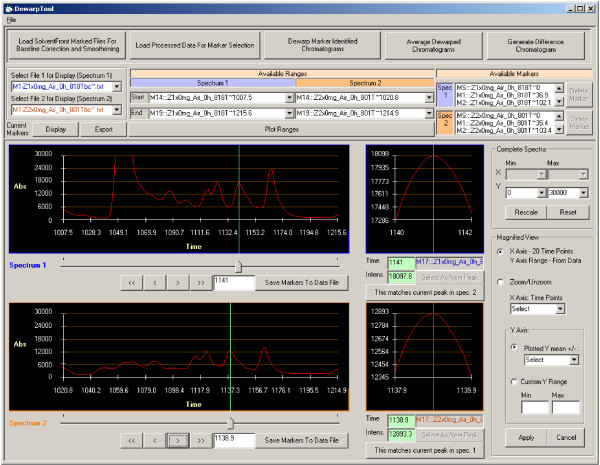
**Main analysis interface of DewarpTool**. The interface allows matching of landmark peaks in a chromatogram to corresponding peaks in a second chromatogram, either a single or an average chromatogram, containing an extensive repertoire of peptide peaks. Magnification views facilitate accurate matching and alignment of peaks.

**Figure 8:**
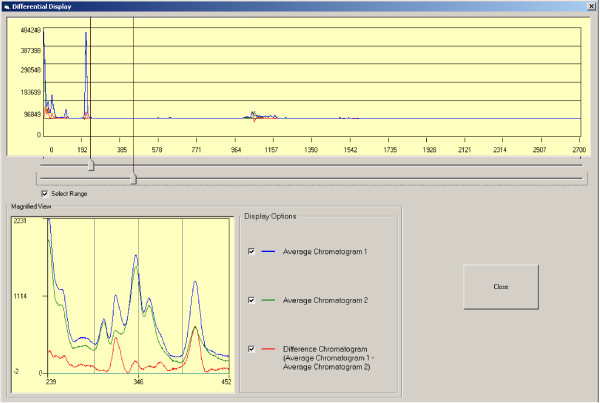
**Differential comparison interface of DewarpTool**. This interface permits comparison of average chromatograms of treatment groups. Sections of average and difference chromatograms can be magnified to examine the reliability of peak alignment and to assess treatment effects.

Peaks common across two or more chromatograms, designated as landmark peaks, are identified (Figure 7), and are uniquely and sequentially numbered. For dewarping, the retention times are compiled for each landmark peak across all chromatograms in which the peak occurs and average retention times are calculated. The landmark peaks are then repositioned to the average retention time by interpolation or removal of data points between the peaks. In our standard and plasma chromatograms, the resolution of the time axis was 0.1 s or 10 points per s. A stretching of landmark peak-to-peak interval in a chromatogram from t1 seconds to t2 seconds (t2>t1) would involve addition of t2-t1 seconds of data, equivalent to (t2-t1)*10 fluorescence data points. Addition of these points is equidistant or isometric, with each new point being added at an interval of (t2-t1)/t1 points, and with the new point being calculated as the average of the two data points between which the insertion is made (to maintain data smoothness). Similarly, shrinking of a landmark peak-to-peak interval from t1 to t2 seconds (t2<t1), involves removal of (t1-t2) seconds of data, equivalent to (t1-t2)*10 data points, at the rate of one data point per every t1/(t1-t2) points. The process of selection of new landmark peaks and dewarping of chromatograms around landmark peaks is iterative with each iteration improving the overall alignment of chromatograms. Dewarped chromatograms from a treatment group can be used to generate average chromatograms within DewarpTool, and visualized with the difference trace (Figure 8) to determine changes that may be of interest, and to serve as a quality check on dewarping.

### Data mining and discriminatory model generation

Both dewarped and non-dewarped chromatographic data were analyzed separately using ClinproTools (version 2.0; Bruker Daltonics, Billerica, MA, USA) for biomarkers. Prior to data import, data preparation and peak selection parameters within ClinproTools were defined as follows: resolution = 100; baseline subtraction by convex hull method; baseline flatness = 0.80; mass range (retention time range) for analysis = 0 – 1200; recalibration with 0.5% or 4.0% maximal peak shift and 30% match to calibrant peaks; signal to noise threshold = 5.0; peak area calculation by zero level integration. Data preparation workflow within ClinproTools consists of baseline correction, spectral normalization based on total ion count (peak count), calculation of a recalibration function for each spectrum to correct for peak shifts, calculation of a total average spectrum using recalibrated peaks, and calculation of areas from individual spectra for peaks represented in the total average spectrum. Recalibration corrects for peak shifts by a process of selection of a number of reference peaks in the average chromatograms and confirmation of occurrence of these peaks in individual chromatograms within a window, as defined by the maximal peak shift parameter. Chromatograms that do not contain a minimum number of reference peaks even after adjusting for the peak shifts are dubbed 'non-recalibratable'. Peaks represented in the total average spectrum are then detected in the individual spectra, their areas are integrated, and the resulting data matrices are used in generation of discriminatory models. Although the chromatographic datasets (both standard analyte chromatograms and plasma chromatograms) were 2700 seconds (45 min) in length, we illustrated the application using the first 1200 seconds (20 minutes) of data that corresponded to the period where the peptides eluted at sufficient peak resolution.

Discriminatory models were built by using the genetic algorithm and support vector machine approaches of the ClinproTools software. Genetic algorithm was applied with a mutation rate = 1.0 (peak substitutions within a model) and recombination rate = 1.0 (peak exchanges between models). Each model was tested for efficiency of classification of experimental samples into correct exposure groups (clean air or EHC-93) and time of recovery groups (0 h and 24 h). The reliability of the models for classification of unknown samples (future predictive capacity) was tested by cross validation using the One-Out, the Random and the K-fold methods. The One-Out method consists of generating a model after leaving one of the chromatograms out and classifying this left-out chromatogram against the model, and repeating this until all chromatograms have been used in classification. For the Random method, 20% of the total chromatograms, randomly selected, are omitted during model building and are then classified against the model generated. This process was repeated 10 times and classification abilities of the generated models were averaged. For the K-fold cross-validation method, the dataset was divided into 12 equal parts, and a part was left out during model building and then classified against the model. The process was iterated until all K parts were used as test data.

### Statistical analysis

Exposures (clean air, EHC-93) and duration of recovery (0, 24 h) as factors were tested for statistical significance of effects by two-way ANOVA, followed by Holm-Sidak's procedure to elucidate the pattern of significant effects (p = 0.05) using Sigma-Stat (Sigma-Stat 3.0, Chicago, Illinois).

## Authors' contributions

RV and SK conceived the dewarping approach described here. SK authored DewarpTool to implement the approach. RV planned and directed animal inhalation exposures. PK developed the chromatographic method for fluorescence analysis of plasma peptides and directed the analysis. SK conducted chromatographic dewarping using DewarpTool and data mining by ClinproTools. SK, PK and RV co-authored the manuscript, while SK led and coordinated the writing of the manuscript. All authors approved the final manuscript.

## Supplementary Material

Additional file 1 DewarpTool Setup Package. Zip file containing DewarpTool installation files. Click here for file

Additional file 2 DewarpTool Installation Instructions. This contains instructions for installation of DewarpTool using the DewarpTool Setup Package (Additional File 1). Click here for file

Additional file 3 DewarpTool User Manual. This is a guide to the use of DewarpTool for dewarping chromatograms. Click here for file
