# Supplementary material for: Data mining of plasma peptide chromatograms for biomarkers of air contaminant exposures
Source: Proteome Sci. 2008 Jan 30;6:6. doi: 10.1186/1477-5956-6-6 (PMC2270821; doi:10.1186/1477-5956-6-6)
Supplement: Additional file 1 — DewarpTool Setup Package. Zip file containing DewarpTool installation files. [file 1477-5956-6-6-S1.zip › DewarpTool Setup Package/Support/LICENSE AGREEMENT.rtf]

LICENSE AGREEMENT

The following terms and conditions enter into effect upon acceptance of the DewarpTool software and documentation for evaluation or use.
Les mondalités de transfert ci-après entrent en vigueur sur acceptation du logiciel DewarpTool et de la documentation pour évaluation ou utilisation.
 
1.  DewarpTool and accompanying documents (the material) are provided free of charge, as is, without warranty of any kind, either expressed or implied, but not limited to the suitability of the material for a particular purpose.  The entire risk as to the quality and performance of the material rests with the receiving organization or person (the user).
DewarpTool et les documents qui l'accompagnent (le matériel) est rendu disponible sans frais, tel quel, sans garantie de quelque nature que ce soit, explicite ou implicite.  Le risque quant à la qualité et à la performance du matériel relève de l'organisation ou la personne récipiendaire (l'utilisateur).

2.  In no event will the copyright holder, the creators, or distributors of the material be liable for any damages arising out of the use or inability to use the material.
Le détenteur des droits d'auteurs, les créateurs, ou les distributeurs ne peuvent être tenus responsables des dommages provenant de l'utilisation ou de l'incapacité d'utiliser le matériel.

3.  The user assumes all responsibilities for the use of the material to achieve its intended results, and for the installation, use and results obtained from said material.
L'utilisateur assume toute responsabilité quant au choix du logiciel ou de la documentation pour réaliser les résultats escomptés et en ce qui concerne l'installation, l'utilisation et les résultats obtenus avec le matériel.

4.  The user may not at any time use the material, either in its original form or in a modified form, for commercial or profit-making purposes, nor sublicense, assign, transfer, sell, lease or grant any right on the material, either in its original form or in a modified form, to other parties.
L'utilisateur ne peut utiliser ou permettre que d'autres utilisent le materiel, sous sa forme originale ou sous une forme modifiée, à des fins commerciales ou dans un but lucratif, ou céder sa licence en sous-traitance, transférer, vendre, louer ou accorder quelque droit que ce soit sur le matériel, sous sa forme originale ou sous une forme modifiée, à une tierce partie.
