# Supplementary material for: Data mining of plasma peptide chromatograms for biomarkers of air contaminant exposures
Source: Proteome Sci. 2008 Jan 30;6:6. doi: 10.1186/1477-5956-6-6 (PMC2270821; doi:10.1186/1477-5956-6-6)
Supplement: Additional file 2 — DewarpTool Installation Instructions. This contains instructions for installation of DewarpTool using the DewarpTool Setup Package (Additional File 1). [file 1477-5956-6-6-S2.pdf]

DewarpTool v. 1.0.
Installation Instructions

1. Download the zip file (DewarpTool Setup Package.zip) containing the installation files from the 'Additional files' section.

2. Create a new folder on your desktop called "DewarpTool".

3. Double-click on DewarpTool Setup Package.zip file to open the zip folder. Copy the contents into the "DewarpTool" folder.

4. Open the "DewarpTool" folder. This should now contain all the necessary installation files as shown below.

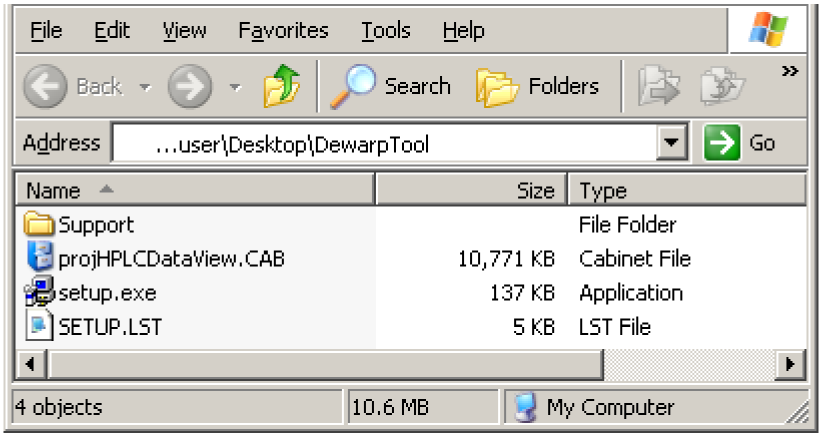

5. Double click on setup.exe to launch the installer. Click 'OK' on the installation start screen (below). Follow on-screen instructions.

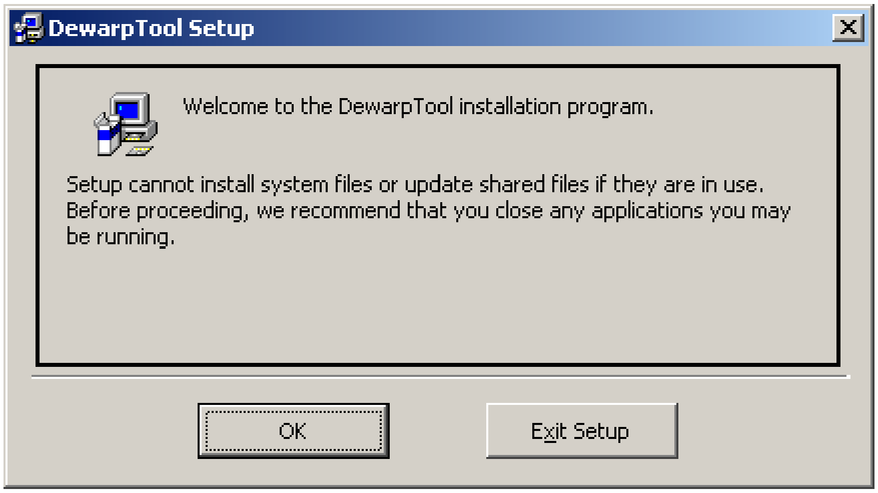

6. A new window is opened up with an install button (shown below). Click on the install button to start installation. Leave the default installation directory unchanged, unless installation in a different location is preferred.

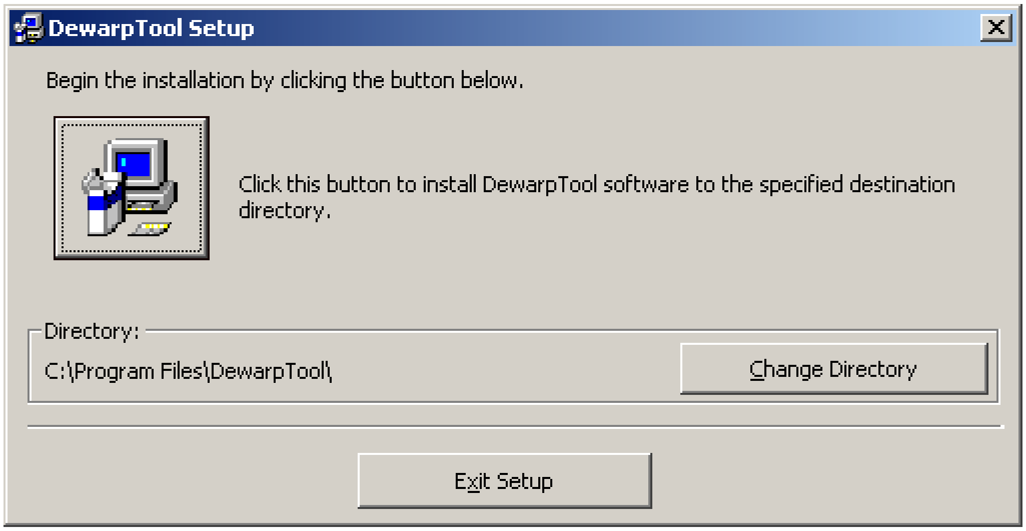

8. Click on ‘Continue’ in the screen below to continue and complete installation.

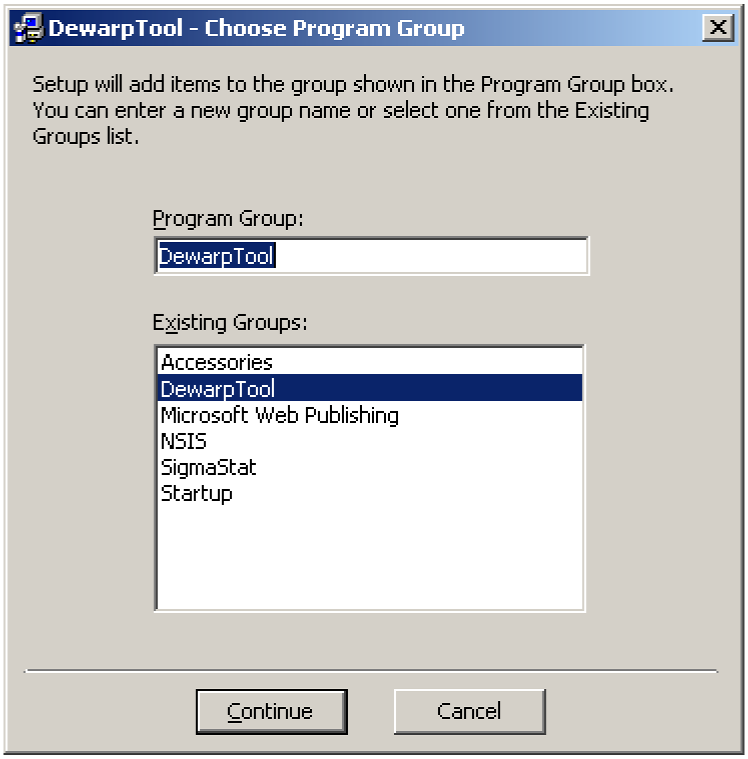

9. After successful installation, the program can be launched by selecting Start → Programs → DewarpTool → DewarpTool.
