# Supplementary material for: Data mining of plasma peptide chromatograms for biomarkers of air contaminant exposures
Source: Proteome Sci. 2008 Jan 30;6:6. doi: 10.1186/1477-5956-6-6 (PMC2270821; doi:10.1186/1477-5956-6-6)
Supplement: Additional file 3 — DewarpTool User Manual. This is a guide to the use of DewarpTool for dewarping chromatograms. [file 1477-5956-6-6-S3.pdf]

DewarpTool Version 1.0.
User Manual

Dewarping workflow starts with chromatographic data preparation involving data export in the ASCII text format using the HPLC software and identification of the solvent front in the exported data using a spreadsheet software, and is followed by baseline correction, data smoothening, selection and matching of landmark (anchor) peaks across chromatograms and dewarping of chromatograms using DewarpTool. DewarpTool also allows chromatogram averaging and differential visualization. However, dewarped chromatographic datasets generated by DewarpTool can be directly used within other applications for analysis of discriminatory biomarker patterns.

Note: The current version (v.1) of DewarpTool works with chromatographic data files of 45 minutes or longer in duration (not including the length of the initial solvent front). A chromatographic data collection rate of 20 data points/second is required.

1. Chromatographic Data Preparation:

i) Export chromatographic data in the ASCII text format. The chromatographic data file when opened using a text editor (e.g. Notepad) should have elution time as the first column and either fluorescence intensity or absorbance (depending on the detector used) as the second column (Fig. 1).

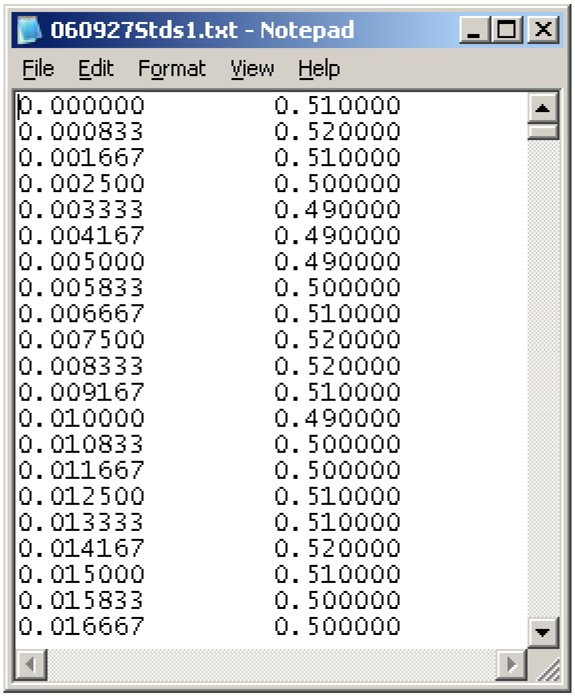

Fig. 1. Raw chromatographic data in the text (.txt) format

ii) Open the data file using a spreadsheet application (e.g. Microsoft Excel). Generate a third column and fill the column cells that correspond to existing data

rows with asterisks. Identify and mark the data point that corresponds to the end of the solvent front (first sample peak in the data series) by a lower-case ‘x’ replacing the asterisk. Save the data file as a new file retaining the text format (.txt file) and editing the filename with an ‘x’ at the beginning and an ‘~’ at the end (e.g. 060920Stds1.txt saved as x060920Stds1~.txt). An example of solvent front identified file is shown below.

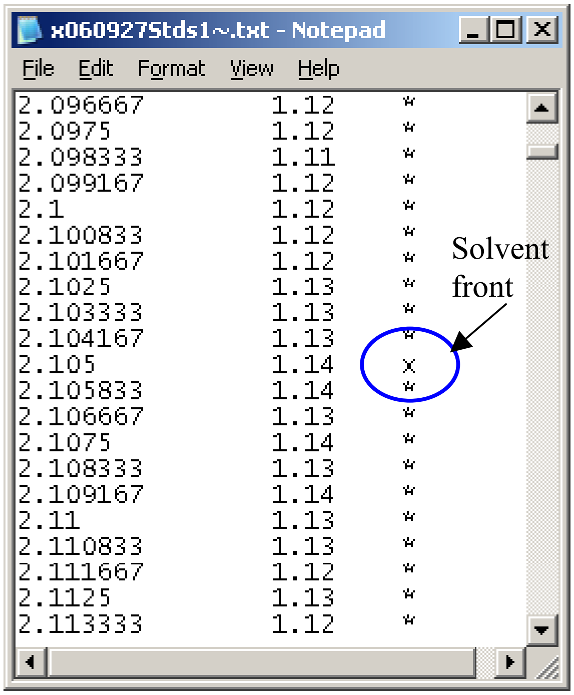

Fig. 2. Chromatographic data after marking the end of solvent front. This is ready for processing within DewarpTool.

2. Baseline Correction and Smoothening

i) Launch DewarpTool by selecting Start, Programs, DewarpTool. In order to import solvent-front marked data files into the application, click on the “Load Solvent Front Marked Files for Baseline Correction and Smoothening” button on the interface (circled blue, Fig 3). This opens up a file selection window.

ii) Using the file selection window, select all solvent-front marked files (having names starting with an ‘x’) and click on ‘Pre-Process and Load Files’ to start baseline correction and smoothening. After baseline correction and smoothening, DewarpTool automatically truncates the solvent fronts (chromatographic section leading up to the first sample peak) and standardizes the lengths of all chromatograms to contain the same number of data points. As the processing of each file is completed, it is automatically saved in the same directory as the source file with a prefix ‘Z’ to the filename (e.g. x060920Stds1~.txt is saved as Zx060920Stds1~.txt) and loaded into the program.

First two of the loaded files are displayed for peak matching and identification of marker peaks. While others are not displayed, they are still available for display and peak selection.

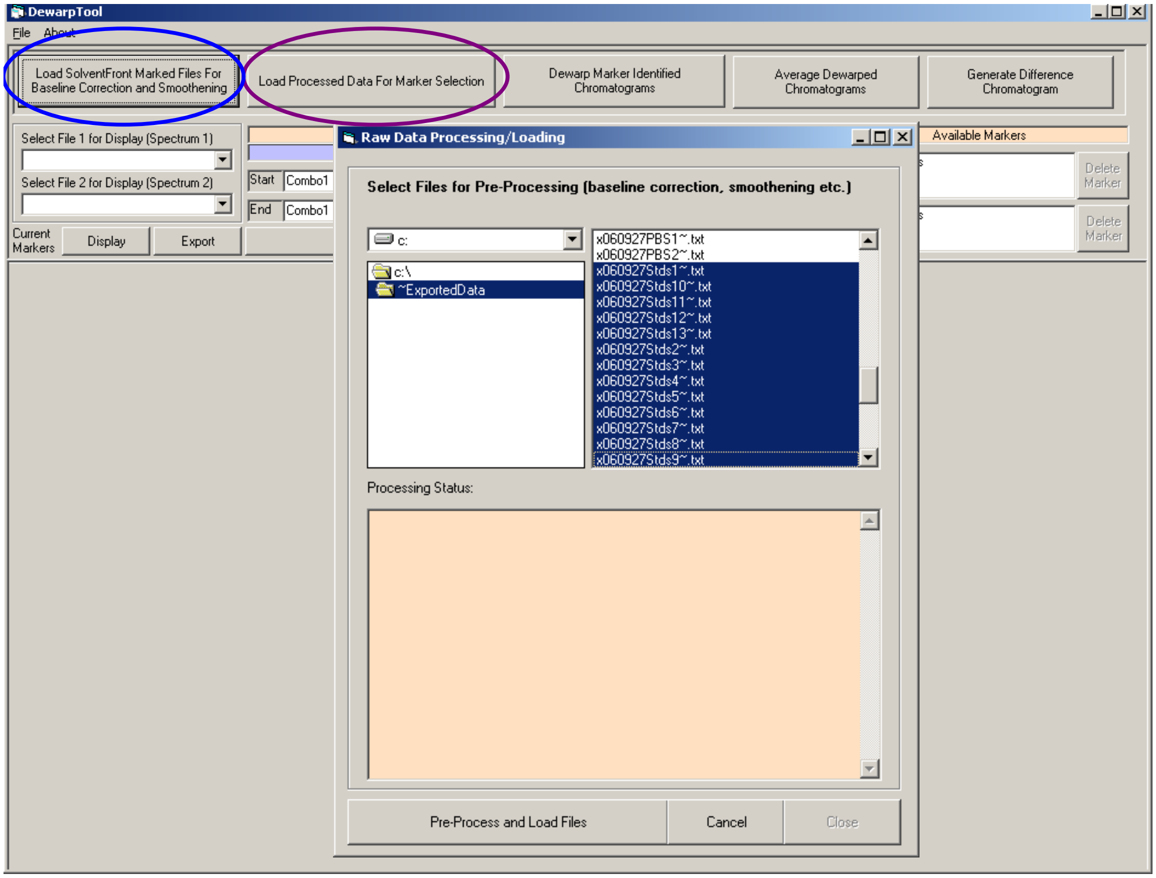

Fig. 3. DewarpTool main interface and the file selection window.

iii) When peak matching and landmark peak identification on baseline corrected and smoothened data are to be continued at a later time, the files can be directly loaded into the program by clicking on the "Load Processed Data for Marker Selection" button (circled purple, Fig. 3) on the main program interface.

3. Selection and Matching of Landmark Peaks ("Markers")

Landmark peaks are peaks common across two or more loaded chromatograms. As they are identified they are uniquely and sequentially numbered within the program. So it is useful to analyze all the chromatograms from an experiment as a set, for the landmark peaks to remain unique and to be correctly identified within the experiment.

i) **Selection and matching of landmark peaks:** Mark a peak as a landmark peak by bringing the slider under a chromatogram (Fig. 4, Region D) to the centre of the peak, either by moving the slider or by using the coarse and fine, forward (>, >>) and backward (<. <<) slider adjustment buttons. Once the slider is centred on a peak, as verified in the magnification view (Fig. 4 Region E), mark it as a new peak by pressing the "Select as New Peak" button under the magnification view. When a matching peak is detected in the second chromatogram, mark it as a matching peak by pressing the 'This Peak Matches Current Peak' button.

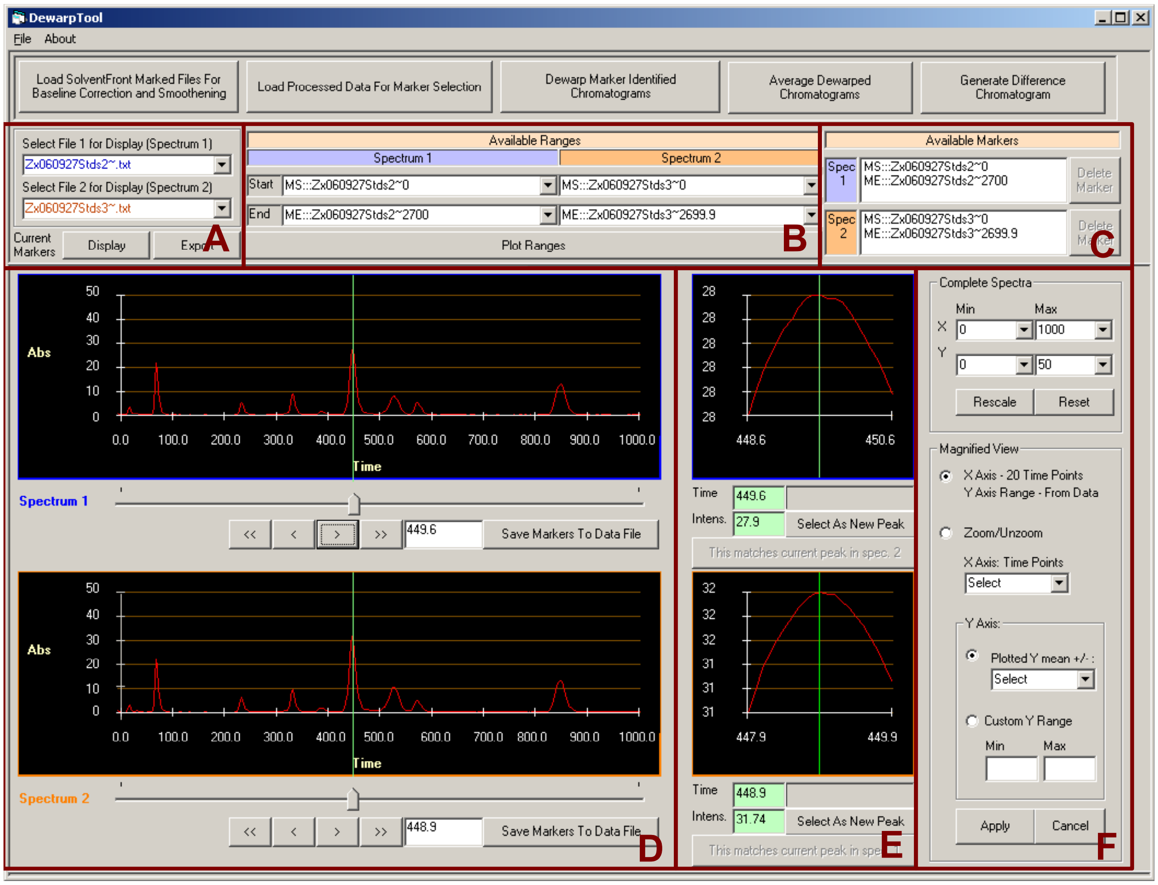

Fig. 4. DewarpTool interface, features enabling landmark peak selection and matching.

Peak selection and matching is also facilitated by the ability to display small chromatographic sections lying between two selected landmark peaks (Figure 4, Region B) and to rescale X axis and Y-axes (Figure 4 Region E).

ii) **Removal of a landmark peak:** If a peak had been erroneously chosen as a landmark peak, it can be deleted by selecting the marker and then clicking on the 'Delete Marker' button beside the peak list (Figure 4, Region C).

iii) **Selection of a new file for landmark peak selection:** Identification and marking of the same landmark peak in multiple chromatograms from an experiment is achieved by retaining a chromatogram containing the landmark peak as the reference chromatogram and displaying the chromatogram in which the marker is to be identified as the second chromatogram (from the file list) (Fig. 4, Region A). After selection of markers in a chromatogram, click on 'Save Marker to Data File' button save the selected landmark peaks to the underlying chromatographic data file. The landmark-peak selected files are saved in the same directory as the source files with a prefix 'M' to their file names.

Both landmark peak selected files (filenames starting with M) and previously dewarped files (filenames starting with DW, see below) can be loaded along with baseline corrected/smoothened chromatograms (filenames starting with Z) using the "Load Processed Data for Marker Selection" button on the DewarpTool main interface, to facilitate landmark peak selection. During iterative dewarping, existing landmark peaks in dewarped chromatograms enable finding additional peaks in the inter-landmark peak regions.

4. Dewarping

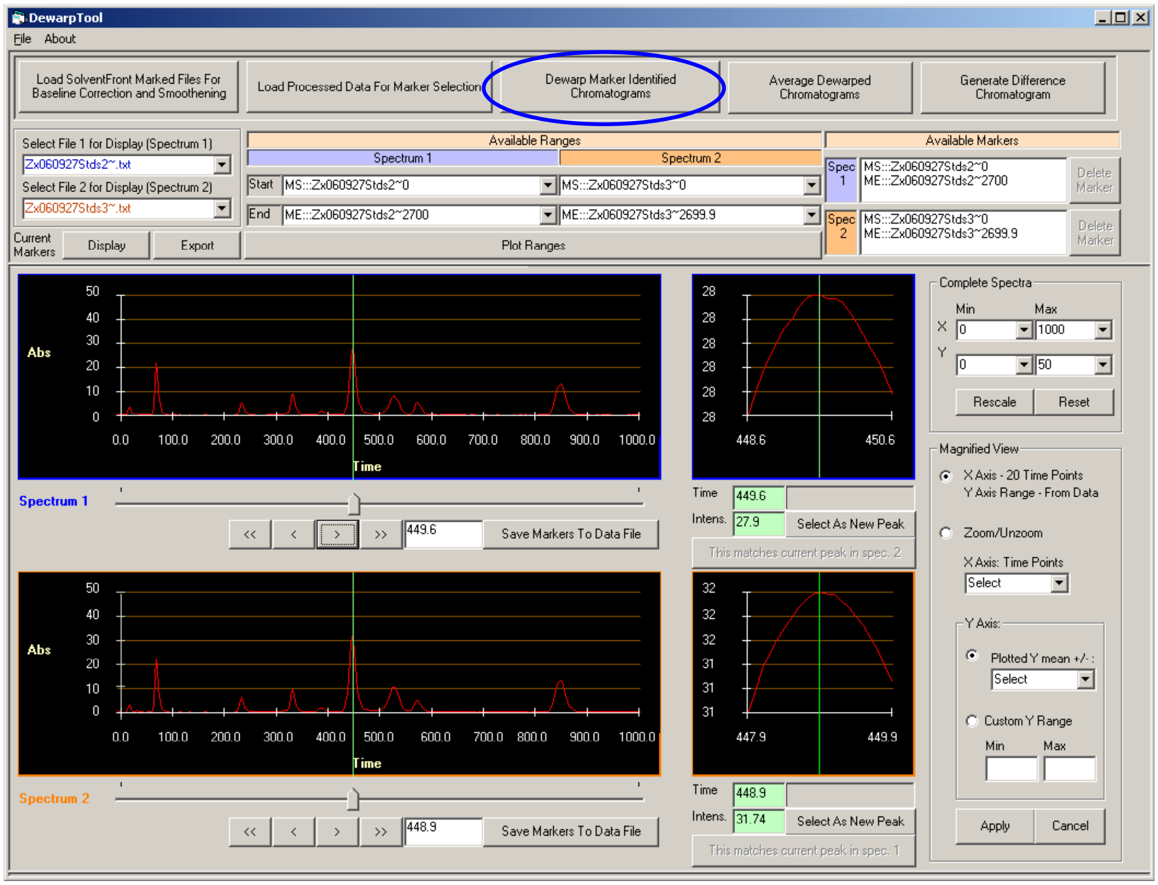

Fig. 5. DewarpTool Main Interface: Option to dewarp currently loaded marker-identified files.

i) Load all landmark peak selected chromatograms from an experiment by clicking on the ‘Load Processed Data for Marker Selection’ button on the main interface, unless they are already loaded into the program confirmed by the file lists.

ii) Click on “Dewarp Marker Identified Chromatograms” (circled blue, Fig. 5) to start compilation of landmark peaks and dewarping. As each chromatogram is dewarped, a new file is generated and renamed with the prefix ‘DW’ and saved in the same folder as the original marked data files.

4. Differential Analysis:

The differential analysis component of the program allows generation of an average chromatogram of all dewarped chromatograms from a treatment group, and visualization of two average chromatograms along with the difference trace to determine changes that may be of significance.

i) Group dewarped chromatograms from different treatment groups into separate folders (e.g. separate folders for control, treatment1, treatment2 chromatograms etc.). Select a set of dewarped chromatograms by clicking on the “Average Dewarped Chromatograms” button (circled blue, Fig. 6) on the main program interface, and selecting the chromatograms using the file selection window.

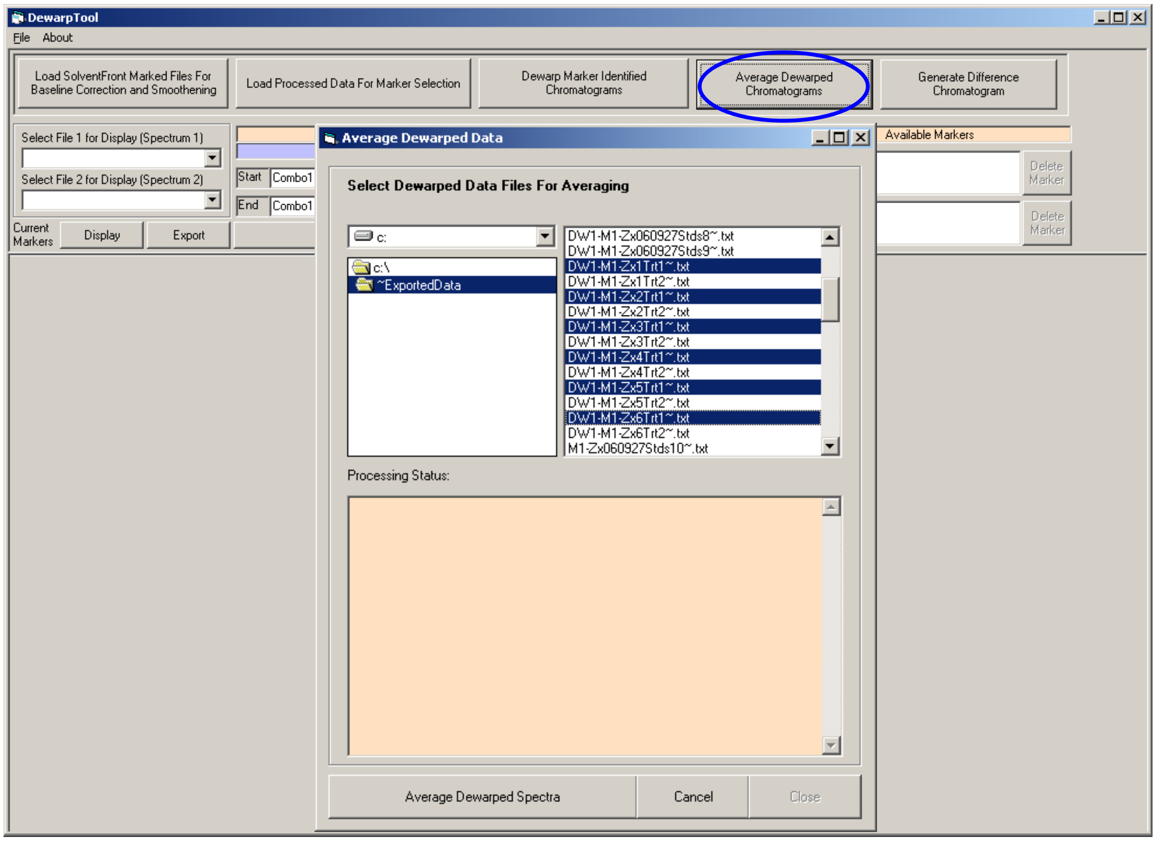

Fig. 6. DewarpTool Main Interface: Selection of dewarped chromatograms for averaging.

ii) Click on the ‘Average Dewarped Spectra’ button (Fig. 6), to generate an average chromatogram for the treatment group. Generate averages for other groups of spectra as desired. An average chromatogram file called ‘AvgChromatogram.txt’ is generated and saved in the same folder as the original dewarped chromatograms.

iii) To generate a difference chromatogram between two average chromatograms click on ‘Generate Difference Chromatogram’ (Fig. 7) and select average chromatograms for differential analysis using the file selection window. Click on ‘Generate Difference Display’ on the file selection window.

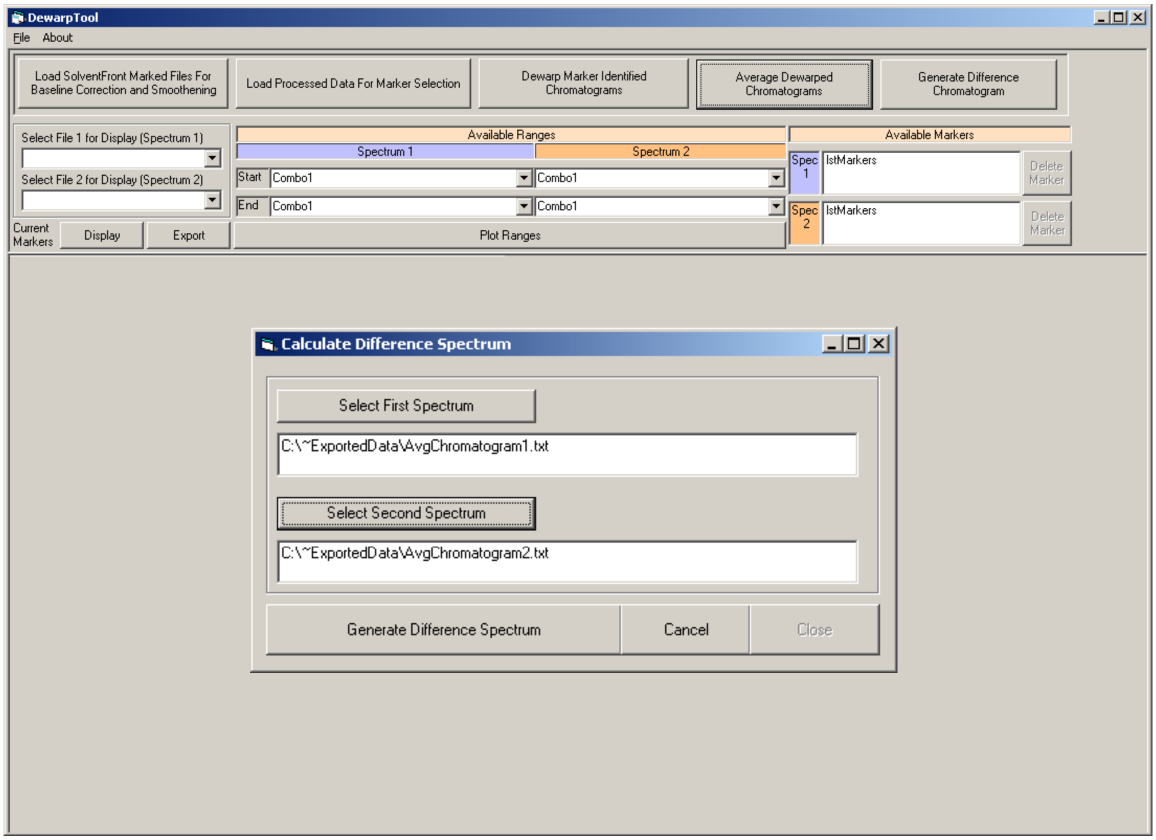

Fig. 7. DewarpTool Main Interface: Selection of averaged chromatograms for differential display.

iv) A new window is opened up with a differential display (Fig. 7) showing the two average chromatograms and the difference trace.

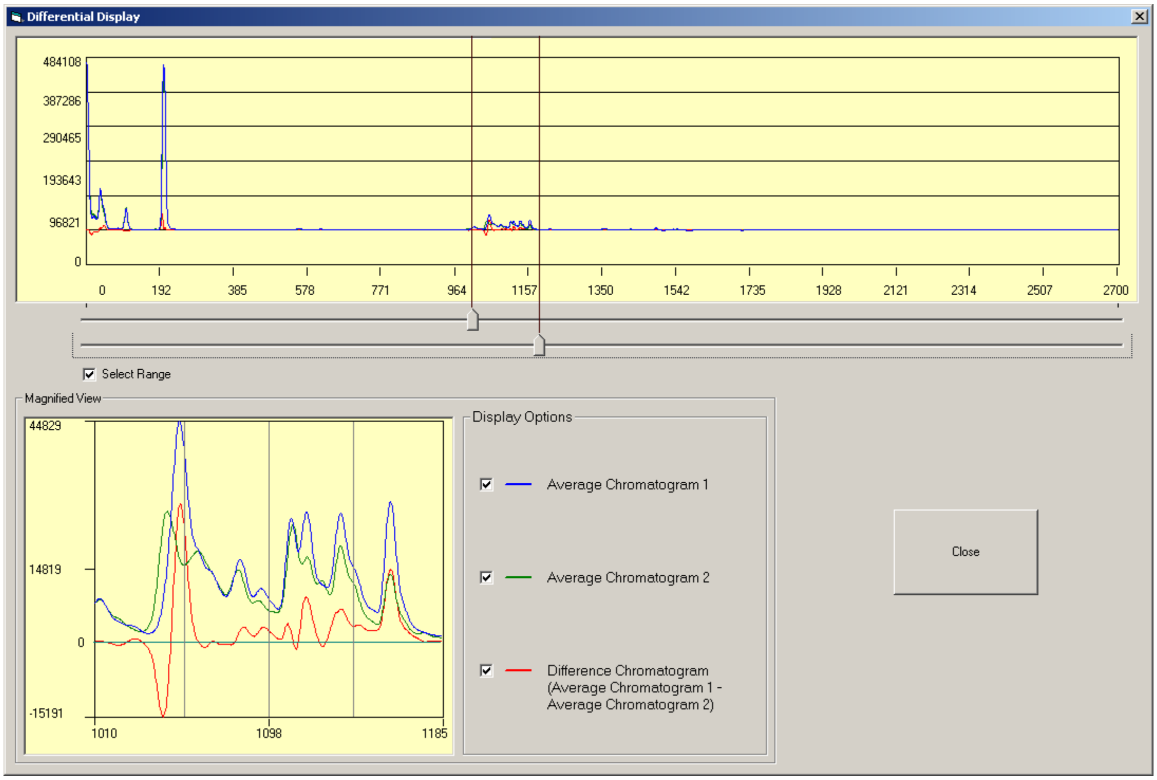

Fig. 8. Differential Display Interface

A magnification view provides a high-resolution visualization of a single peak or a small segment of the time axis, as defined by using the sliders on the full chromatogram display.

The dewarped chromatograms (filenames starting with 'DW') can also be used within other applications for mining of discriminatory biomarker patterns after adjustments to the file format as may be required by external applications.
